# Yiqihuoxue Formula Activates Autophagy and Offers Renoprotection in a Rat Model of Adenine-Induced Kidney Disease

**DOI:** 10.1155/2019/3423981

**Published:** 2019-12-24

**Authors:** Chen Hui Xia, Xue Ting Han, Xueqin Zhang, Ze Bing Zhu, Jing Guo, Hai Lan Cui, Han Xue Jiang, Wei Jun Huang, Guo Zi Chen, Yu Ning Liu, Wei Jing Liu

**Affiliations:** ^1^Key Laboratory of Chinese Internal Medicine of Ministry of Education and Beijing, Dongzhimen Hospital Affiliated to Beijing University of Chinese Medicine, Beijing, China; ^2^Renal Research Institute of Beijing University of Chinese Medicine, Dongzhimen Hospital Affiliated to Beijing University of Chinese Medicine, Beijing, China; ^3^Shenzhen Hospital Affiliated to Beijing University of Chinese Medicine, Shenzhen, Guangdong, China

## Abstract

Chronic kidney disease (CKD) is a worldwide health problem for which effective therapeutic methods are still lacking. Traditional Chinese medicine (TCM) has been indicated as an effective alternative treatment for kidney disease. In this study, a clinically effective therapy, yiqihuoxue (YQHX) formula, was administrated to adenine-induced kidney disease rats for 6 weeks. We found that the adenine rats displayed a significant reduction in renal function as evidenced by the increased levels of serum creatinine (Scr), blood urea nitrogen (BUN), and 24-h urinary albumin level, which were attenuated by the YQHX treatment. The glomerulosclerosis, interstitial fibrosis, arteriolosclerosis, interstitial inflammation, and tubular dilatation were reversed by the YQHX treatment in the adenine rats. Furthermore, the hepatic damage characterized by increased levels of aspartate aminotransferase and alanine aminotransferase and inflammatory cell infiltration was improved by YQHX. In addition, the number of apoptotic cells in the adenine rats was obviously reduced by the YQHX treatment as manifested by the lower expression level of cleaved caspase-3 protein. Moreover, the YQHX treatment downregulated the expression levels of fibronectin, type I collagen, *α*-smooth muscle actin, and TGF-*β*1 in the adenine rats. Furthermore, autophagy was activated by the YQHX treatment, which manifested as an increased LC3-II and Beclin-1 expression levels and a decreased p62 level. In conclusion, the YQHX formula might retard the progression of kidney disease by activating autophagy.

## 1. Introduction

The incidence of chronic kidney disease (CKD) has increased annually, and the deterioration of the air environment has provided a hotbed for its rapid development [1]. Especially in developing countries, the deterioration of the air environment, the insufficiency of medical resources, and the lack of awareness of chronic diseases have all been contributing to the prevalence of CKD. In China, the incidence of CKD is up to 10.8% and the awareness rate is only 12.5% [2]. CKD is characterized by low creatinine clearance, high blood urea nitrogen level, and hyperuricemia, concomitant with hypoproteinemia and anemia, electrolyte disturbance, and mineral bone metabolism disorder, as well as secondary hyperparathyroidism [3]. Along with the complex and diverse pathogenic features and clinical symptoms and complications, its molecular biological mechanisms also remain complicated, making it difficult to identify effective treatments.

The yiqihuoxue (YQHX) formula is a traditional Chinese medicine (TCM), which is capable of promoting blood circulation and removing blood stasis in the theory of TCM. The decoction is extracted from 8 distinct herbs, including Radix Astragali seu Hedysari, Radix Cyathulae, Fructus Aurantii Immaturus, Rhizoma Polygoni Cuspidati, Rhizoma Sparganii, Rhizoma Curcumae, Eupolyphaga Seu Steleophaga, and Hirudo (Table 1). Its aqueous extract has been clinically used for decades and confirmed to be an effective therapeutic method for the treatment of many types of CKD owing to its excellent properties of increasing the creatinine clearance and reducing the urinary protein excretion. By its network pharmacology, as a TCM formula, the YQHX decoction may function by delivering the beneficial effects of its multiple compounds, acting on multiple targets and pathways [4]. However, the exact mechanism, thereby, the YQHX formula could contribute to the clinical improvement of CKD is unclear.

Accumulating evidence indicates that fibrotic and apoptotic responses play significant roles in CKD progression and development [5–7]. Autophagy activation is reported to have protective effects against diverse renal cell injuries, such as hypoxia, ischemia/reperfusion, oxidative stress, and end-stage renal fibrosis [8–10]. In addition, proteinuria, as a damaging factor, can result in renal tubular atrophy and interstitial fibrosis [11, 12]. In our previous study, we confirmed that autophagy activation could protect renal tubular epithelial cells (TECs) from urinary protein-induced injury [13]. Thus, on the basis of the therapeutic effect of reducing urinary protein excretion, the YQHX formula was tested to investigate whether it could further ameliorate the damage caused by large amounts of proteinuria by activating the autophagic pathway.

## 2. Materials and Methods

### 2.1. YQHX Formula

YQHX is a decoction of 8 Chinese herbal medicines at a ratio of 10 : 5 : 5 : 5 : 4 : 4 : 4 : 1 (Table 1). Aqueous extract granules were prepared in our hospital (Dongzhimen Hospital, Beijing). Before treatment, the granules were dissolved with boiled ddH_2_O to achieve a concentration of 1 g/ml.

### 2.2. Animal and Treatment Protocol

Adult male Sprague Dawley (SD) rats, with body weights of 230–240 g, were purchased from the Beijing Vital River Laboratory Animal Technology Co., Ltd. (Beijing, China; permission no. SYXK [Beijing] 2017–0022). All animal experimental studies were conducted in accordance with the Guidelines on Human Use and Care of Laboratory Animals for Biomedical Research, published by the National Institutes of Health (NIH publication no. 85–23, revised in 1996). The experimental protocol was approved and performed by the Ethical Animal Committees of Dongzhimen Hospital, Beijing University of Chinese Medicine.

The rats were raised in the Animal Research Institute of Dongzhimen Hospital, at a constant temperature (22–25°C) and humidity (40%–70%). All the animals were kept on a 12-h light/dark cycle and had free access to food and water ad libitum. After 1 week of acclimatization, the rats were divided randomly into 3 groups as follows: (1) normal control group (*n*=8), (2) adenine group (*n*=8), and (3) YQHX-treated group (adenine + YQHX, *n*=8). In the adenine group and the treatment group, adenine (0.75% w/w) was intragastrically administered for 4 weeks. The rats in the YQHX group received 12 g/kg/d dose (translating by the conversion factor 6.25 with the adult dose 114 g/d in clinical practice) of the YQHX granule extract orally for 6 weeks after adenine administration, while the rats in the control group were given the same volume of saline solution. Three days before the rats were killed, the 24-h urine of all the rats were collected and centrifuged at 1500 r/min for 5 min. The animals were sacrificed by exsanguination from the abdominal aorta after anesthesia with 0.3% pentobarbital (1 ml/100 g). Blood was collected into sterile tubes for centrifugation at 4°C and 3000 rpm for 15 min. The left kidney tissue was fixed with 4% paraformaldehyde (pH 7.4) and embedded in paraffin for histological staining, while the right kidney was stored in liquid nitrogen for western blotting analysis.

### 2.3. Biochemical Analysis

The levels of serum creatinine (Scr), blood urea nitrogen (BUN), aspartate aminotransferase (AST), and alanine aminotransferase (ALT) were measured using serum detection kits (C011-2, C013-1-1, C0010-2-1, and C009-2-1; Nanjing Jiancheng Bioengineering Institute, Nanjing, China) in accordance with the manufacturer's instructions. The 24-h urine albumin quantitative measurement was performed with a rat albumin enzyme-linked immunosorbent assay kit (ab108789, Abcam).

### 2.4. Histological Examination

The paraffin-embedded kidneys and livers were cut into 3-*μ*m sections, dewaxed for 3 × 15 min within the xylene reagent tank, and then rehydrated using ethyl alcohol in a degradation concentration. Hematoxylin-eosin (H&E) and periodic acid-Schiff (PAS) staining were performed to observe pathological changes. Masson's trichrome staining (Masson) was applied to each section as reported for the fibrosis evaluation. Images were captured using a Zeiss optical microscope with the ZEN 2.3 (blue edition) image capture software.

Glomerulosclerosis index (GSI) was evaluated by mesangial expansion and sclerosis as described previously [14]. Fifty glomeruli per animal on PAS-stained kidney section (×200 magnification) were assessed and graded from 0 to 3 by a semiquantitative score displayed in Table 2. The GSI for each animal was calculated as a mean value of all glomerular scores obtained. Tubulointerstitial indexes were assessed with the method mentioned by Véniant et al. [15]. The parameters of interstitial fibrosis, arteriolosclerosis, interstitial inflammation, and tubular dilatation were determined, respectively, using a semiquantitative scoring method on Masson-, PAS-, and HE-stained sections at a magnification of ×200. Ten fields per kidney were assessed by assigning a score from 0 to 3 (0 = no abnormality, 1 = mild, 2 = moderate, and 3 = severe) according to the severity degree displayed in Table 2. The liver inflammation was assessed by the method of Ishak et al. [16] on HE-stained sections at a magnification of ×100. The semiquantitative criteria of lesion degrees are displayed in Table 2. Ten kidney fields were randomly chosen from each rat in the group of control (*n*=8), adenine (*n*=8), and YQHX (*n*=8). And the histologic analysis was performed by two independent investigators in a blind fashion.

### 2.5. Immunohistochemistry

After antigen retrieval using a microwave oven at 95°C, soaking in sodium citrate (pH 6.0) for 20 min, and cooling at room temperature (approximately 25°C), the sections were treated with 3% hydrogen peroxide for 20 min to block the endogenous peroxidase. After washing with PBS, the blocking serum was applied for 30 min. The sections were incubated with the anti-TGF-*β* antibody (ab92486, Abcam, 1 : 50), anti-collagen-I antibody (ab34710, Abcam, 1 : 100), anti-*α*-SMA antibody (55135-1-AP, Proteintech, 1 : 100), and anti-caspase-3 antibody (ab2302, Abcam, 1 : 50) at 4°C overnight. The secondary antibody incubation was performed with an enhanced enzyme-labeled goat anti-mouse/rabbit IgG polymer reagent (PV 9001/PV9002, Beijing Zhongshan Jinqiao Biotechnology Co., Ltd., Beijing). The reaction was visualized with 3,3′-diaminobenzidine, and the nuclei were counterstained with hematoxylin. The reproducibility test was conducted using the same protocol in multiple, randomly selected specimens. All the images were captured as previously described, under ×200 or ×400 magnification. Five animals were randomly selected from each group, and 15 pictures were taken randomly from each animal slice. The mean optical density (MOD) of each of the pictures of TGF-*β*, *α*-SMA, and Col-I staining was calculated using the Image-Pro Plus 6.0 software (Media Cybernetics, USA). The MOD for each animal was calculated as a mean value of the total MOD measured from the 15 pictures per rat. Meanwhile, the expression level of the three proteins was also assessed by the lesion degree (presented in Supplementary [Supplementary-material supplementary-material-1]) according to Gadola et al. [14]. The level of apoptosis of the kidney indicated by the expression level of caspase-3 was calculated from the ratio of apoptotic cells to the total renal cells.

### 2.6. Immunofluorescence

The paraffin-embedded kidney tissues were cut into 3-*μ*m sections, dewaxed, rehydrated, and antigen retrieved as previously described. To increase the cell membrane permeability to antibodies, 0.2% Triton 100X (Solarbio, T8200) was extracted. After incubation with the blocking serum for 1 hour at room temperature (approximately 25°C), the sections were stained with the anti-LC3B antibody (ab51520, Abcam, 1 : 1000) and the anti-p62 antibody (ab56416, Abcam, 1 : 200) at 4°C overnight, followed by the donkey anti-rabbit/mouse IgG (H + L) highly crossadsorbed secondary antibody (A-21206/A-21203, Invitrogen, 1 : 1000). The nuclei were counterstained with 4′6-diamidino-2-phenylindole. Image acquisition and processing were performed as previously described. The immunofluorescence staining was assessed with a semiquantitative scoring method by two independent observers unware of the treatment received by each group.

### 2.7. Western Blotting Assay

Total protein lysate extraction from the renal cortical section of the kidney tissue was performed using a radioimmunoprecipitation assay lysis buffer (C1053, Applygen, Beijing) with a protease inhibitor (KGP603, Nanjing KeyGen Biotech Co., Ltd.), and the protein lysate concentration was determined using a BCA protein assay kit (P1511-2, Applygen) based on the manufacturer's instructions. Equal amounts of kidney cortex protein were loaded into 12%/8% SDS-PAGE and transferred into PVDF membranes. After being blocked with 5% nonfat milk for 1 h at room temperature, the membranes were incubated with primary antibodies on the bed temperature incubator at 4°C overnight. The primary antibodies were as follows: LC3B (ab51520, Abcam, 1 : 2000), p62 (ab56416, Abcam, 1 : 1000), Bcl-2 (ab194583, Abcam, 1 : 500), TGF-*β* (ab92486, Abcam, 4ug/ml), FN (ab2413, Abcam, 1 : 1000), *α*-SMA (55135-1-AP, Proteintech, 1 : 1000), and Col-I (ab34710, Abcam, 1 : 1000). Then, the PVDF membranes were incubated with HRP-conjugated second antibodies (SA00001-1 and SA00001-2, Proteintech, 1 : 5000). *β*-Actin (66009-1-Ig, Proteintech, 1 : 1000) was used as the loading control for protein expression. The chemiluminescence signals were visualized using the ECL Plus western blotting detection reagents (B500021, Proteintech). Densitometric analysis of the band optical density was performed using the ImageJ 1.51K software (National Institutes of Health, USA).

### 2.8. Statistical Analysis

Data were expressed as mean ± SD. Multiple comparisons between the groups were performed using one-way analysis of variance. A *p* value of <0.05 was considered statistically significant. GraphPad Prism v6.0 was applied for the analyses.

## 3. Results

### 3.1. YQHX Treatment Preserved Renal and Hepatic Function in the Adenine Rats

**Figure 1:**
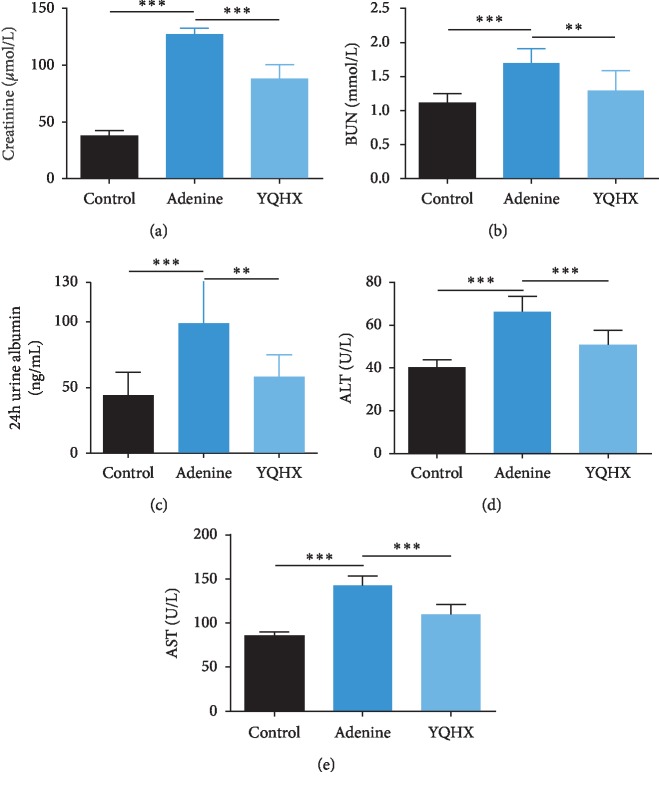
YQHX treatment improved the renal and hepatic function of the adenine rats. (a–c) After treatment with the YQHX formula, the levels of serum creatinine, BUN, 24-h urine albumin in the adenine group were reduced. (d and e) The levels of serum ALT and AST were assayed and found to be reduced in the YQHX group as compared with the adenine group. Adenine group: adenine-induced kidney disease rat. *n*=8 per group. ^*∗∗*^*p* < 0.01 and ^*∗∗∗*^*p* < 0.001.

Renal function parameters including Scr and BUN were assessed, as well as the 24-h urine albumin. Compared with the control group, the adenine group had significantly elevated Scr and BUN levels, which were obviously reduced by the YQHX treatment (Figures 1(a) and 1(b)). Moreover, 24-h urine albumin level was high in the adenine group but decreased in the YQHX group (Figure 1(c)). The data mentioned above indicated that the model of kidney disease was established successfully, and YQHX treatment had the effect of improving renal function in the adenine rats. To investigate the adenine-induced hepatic damage, the parameters including ALT and AST were assessed. The results show that the increased levels of the both parameters were decreased by the YQHX treatment, indicating the protective effects of the formula on liver function.

### 3.2. YQHX Treatment Ameliorated Renal and Hepatic Pathological Injury in the Adenine Rats

**Figure 2:**
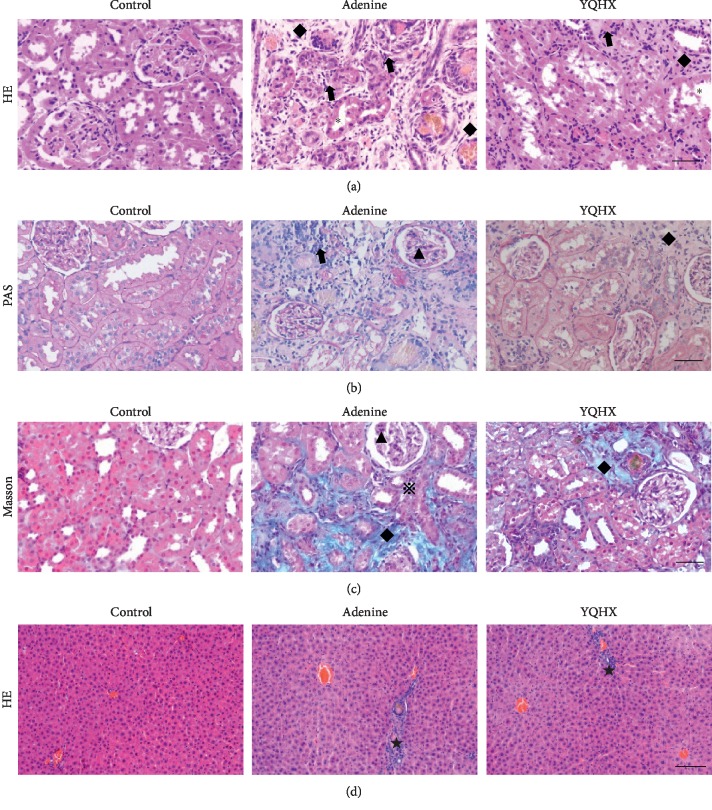
Morphological examination of the kidney and liver about the effect of the YQHX treatment on adenine rats. (a–c) Kidney HE, PAS, and Masson staining: the control group shows normal tubular and glomeruli architecture. The adenine group shows glomerulosclerosis (▲), interstitial fibrosis (◆), arteriolosclerosis (※), interstitial inflammation (←), and tubular dilatation (^*∗*^). The YQHX group shows amelioration of the renal structure. All the images are shown at a magnification of ×200, scale bar = 50 *μ*m. (d) Liver HE staining: portal inflammation (★) was slightly ameliorated by YQHX. Images are shown at a magnification of ×100, scale bar = 100 *μ*m. Adenine group, adenine-induced kidney disease rat; YQHX, yiqihuoxue group.

In the present study, H&E, PAS, and Masson were performed to further evaluate the effects of YQHX on the morphological changes of kidney tissues. In the H&E and PAS staining, compared with the control group, the model group showed significant damage, including diffuse glomerulosclerosis and interstitial fibrosis, arteriolosclerosis, infiltration of numerous inflammatory cells, and obvious renal tubular dilation, which were significantly improved by the YQHX treatment (Figures 2(a), 2(b) and Table 3). Masson's trichrome staining revealed that renal fibrosis was significantly reduced in the YQHX group as compared with the adenine group (Figure 2(c) and Table 3). Besides, livers of the rats were examined by H&E staining and the result showed that the liver tissue treated with adenine was infiltrated by massive inflammatory cells in the portal area. Notably, the YQHX group has shown an ameliorative effect as reduced inflammation cell infiltration, indicating the protective effects of the formula towards hepatic injury induced by adenine (Figure 2(d) and Table 3).

### 3.3. YQHX Attenuated Renal Fibrosis in the Adenine Rats

**Figure 3:**
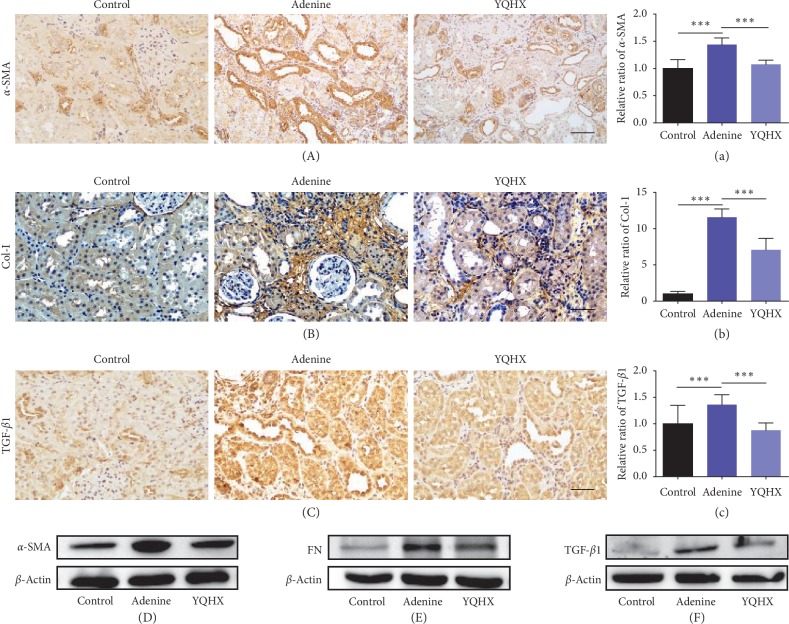
YQHX treatment reduced the expression of renal fibrosis-relative proteins. (a–c) Immunohistochemical staining of *α*-SMA, Col-I, and TGF-*β*1 in the control, adenine, and YQHX groups. The number of rats per group was 5, and 10 pictures were taken from each rat. Scale bar: 50 *μ*m. Original magnification: ×200. The optical intensity of the abovementioned proteins was measured. ^*∗∗∗*^*p* < 0.001. (d–f) Western blotting analysis results showing increased protein levels of *α*-SMA, FN, and TGF-*β*1 in the adenine group, which were decreased by the YQHX treatment. Adenine group, adenine-induced kidney disease rat; YQHX, yiqihuoxue group.

To further observe the molecular biological changes of the kidney tissue, we examined the expression levels of fibrotic markers. Consistent with the result of Masson's staining, an increased deposition of collagen manifested as increased collagen-I level and the *α*-smooth muscle actin (*α*-SMA) level was upregulated in the adenine rats, both of which were reversed in the YQHX treatment group (Figures 3(a) and 3(b)). A known marker of fibrosis, transforming growth factor-1 (TGF-*β*1), was examined using immunohistochemical staining and western blotting. With both methods, TGF-*β*1 showed an increased level in the adenine group as compared with the control group, which was improved by the YQHX treatment (Figures 3(c) and 3(f)). And the same results were observed by the lesion-based scoring method (Supplementary [Supplementary-material supplementary-material-1]). In addition, the western blot was applied to detect the expression levels of a-SMA and fibronectin (FN). As shown in Figures 3(d) and 3(e), the expression levels of *α*-SMA and FN were all upregulated in the adenine group and reduced in the YQHX treatment group.

### 3.4. YQHX Ameliorated Apoptosis in Kidney Tissue in the Adenine Rats

**Figure 4:**
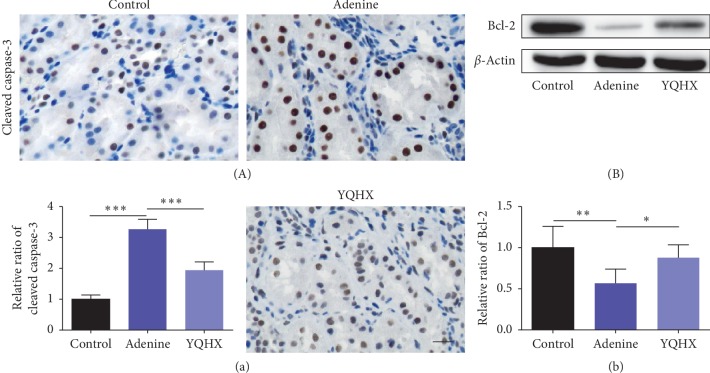
YQHX treatment attenuated renal apoptosis in the adenine rats. (a) Immunohistochemical staining was performed to detect renal apoptosis. The level of apoptosis of the kidney was calculated from the ratio of apoptotic cells to the total renal cells. The YQHX treatment reduced renal apoptosis in the adenine group. The number of rats per group was 5, and 10 pictures were taken from each rat. Scale bar: 20 *μ*m. Magnification: ×400. ^*∗∗∗*^*p* < 0.001. (b) Western blotting and the Bcl-2 level are shown. ^*∗*^*p* < 0.05 and ^*∗∗*^*p* < 0.01. *n*=6 per group. Adenine group, adenine-induced kidney disease rat; YQHX, yiqihuoxue group.

Apoptosis was reported to have important relationships with fibrosis. In the present research, we further detected the apoptosis level in the kidney tissue by immunohistochemical analysis of the well-recognized marker of apoptosis, cleaved caspase-3, and western blot examination of the antiapoptotic protein Bcl-2. The high expression level of cleaved caspase-3 in the adenine group was significantly reversed by the YQHX treatment (Figure 4(a)). We found an increased Bcl-2 level in the YQHX treatment group as compared with the adenine group (Figure 4(b)). These data indicate that YQHX has a beneficial effect on renal apoptosis amelioration.

### 3.5. YQHX Activated Autophagy by Autophagic Induction in the Adenine Rats

**Figure 5:**
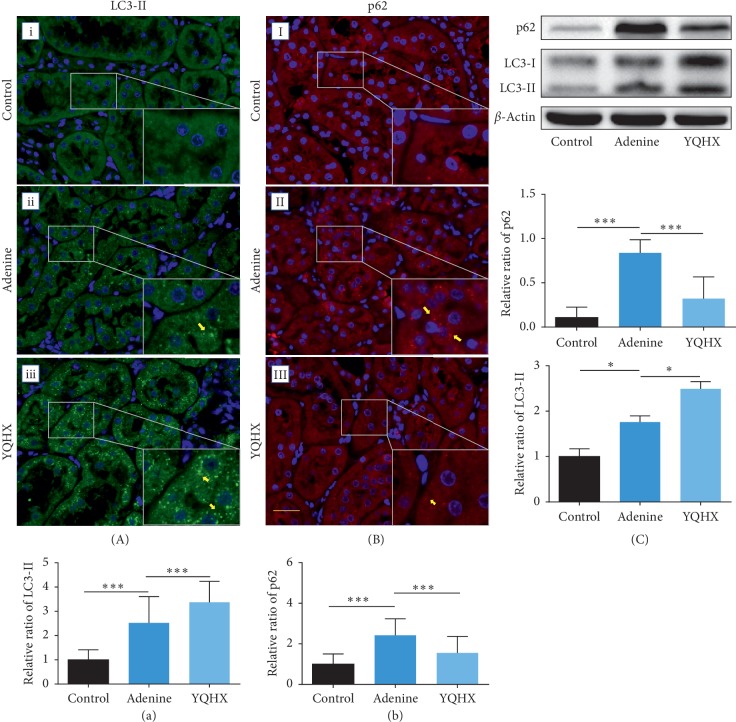
YQHX treatment improved the activation level of renal autophagy in the adenine rats. (a, b) Immunofluorescence staining was applied to detect the changes in LC3-II and p62 expression levels in the control, adenine, and YQHX groups. The puncta with green color indicates the autophagosomes represented by LC3-II, while the red ones indicate p62. The number of rats per group was 5, and 10 pictures were taken randomly from each rat. Scale bar: 20 *μ*m. Magnification: ×400. ^*∗∗∗*^*p* < 0.001. (a, b) Semiquantitative analysis using the scoring method. (c) Results of the western blotting analysis of p62 and LC3-II. The high expression level of p62 was downregulated by the YQHX treatment. The higher LC3-II level in the adenine group was increased, with further increased by the YQHX treatment. ^*∗*^*p* < 0.05 and ^*∗∗∗*^*p* < 0.001. *n*=6 per group.

**Figure 6:**
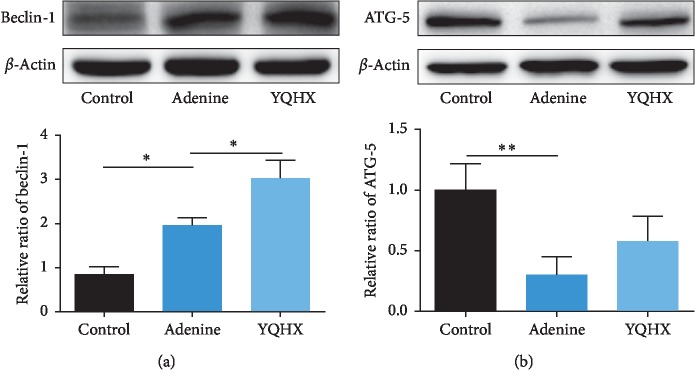
YQHX treatment improved the induction of autophagy in the adenine rats. (a, b) Western blotting analysis results showing the expression levels of Beclin-1 and ATG-5. The Beclin-1 expression in the adenine group was upregulated by the YQHX treatment. The lower ATG-5 level in the adenine group shows an uptrend by the YQHX treatment. ^*∗*^*p* < 0.05 and ^*∗∗*^*p* < 0.01. *n*=6 per group.

We detected LC3-II and p62 expression levels in the rat kidneys in all the groups by immunofluorescence staining. As shown in Figure 5(a), the expression level of LC3-II was slightly increased in the adenine group as compared with the control group. Prominently higher LC3-II levels were found in the YQHX group than in the adenine group. Meanwhile, the expression of the autophagy substrate p62 was significantly increased in the adenine group and reduced by the YQHX treatment (Figure 5(b)). To further confirm the results, western blot analysis was performed to observe the expression levels of both LC3-II and p62 in the kidney tissues and the results were obviously consistent with the immunofluorescence staining (Figure 5(c)). Moreover, to examine the autophagic induction activity, the expression levels of Beclin-1 and ATG-5 were examined. The Beclin-1 expression showed the same pattern as the LC3-II expression level (Figure 6(a)). However, ATG-5 showed a lower level in the adenine group but was restored in the YQHX group (Figure 6(b)).

## 4. Discussion

CKD is a worldwide health problem, characterized by a prominent histomorphological change of glomerular sclerosis and tubular fibrosis in response to a series of injuries. Thus, it is important to find a therapeutic approach to restore renal function. In the present study, the YQHX formula, a clinical effective medication, showed a significant renal protective effect in adenine rats by decreasing the concentrations of Scr, BUN, and 24-h urine albumin. Furthermore, histological analysis revealed an obvious restorative function of the YQHX formula in ameliorating the renal damage such as glomerulosclerosis, interstitial fibrosis, arteriolosclerosis, interstitial inflammation, and tubular dilatation. However, the histological staining of the kidney was only conducted on the left kidneys. While several reports mentioned that the two kidneys responded differently towards the injury [17]. Thus, the histological changes of the right kidneys need further studies to confirm. In addition, the YQHX treatment showed a protective effect towards liver function by lowering the levels of ALT and AST. Besides, the inflammation cell infiltration induced by adenine was also ameliorated by the YQHX treatment, indicating its antiinflammation effects.

Renal fibrosis is related to the diverse production and release of bioactive mediators and metabolites, which were shown to be toxic to TECs [18]. However, some researchers also indicated that TECs not only are victims of various damage factors but also play the role of fibrogenic cells by releasing a series of growth factors such as transforming growth factor-*β* (TGF-*β*), connective tissue growth factor, and vascular endothelial growth factor [19]. The TGF-*β* signaling pathway has been regarded as a distinguishing marker of tubulointerstitial fibrosis, which contributes to the phenotypic conversion program of epithelial-mesenchymal transition (EMT) by gaining the mesenchymal features (including *α*-smooth muscle actin, interstitial matrix component type-I collagen, and FN) [20–22]. Furthermore, owing to the increase in the fibrotic factors, an excessive deposition of the extracellular matrix (ECM) occurred, consisting of some collagens and fibrotic proteins (Col-I, Col-III, FN, etc.) in the tubulointerstitial area [23]. Thus, in the present study, the levels of TGF-*β*, as well as *α*-SMA, Col-I, and FN were detected. Notably, the results showed that the elevated levels of fibrotic markers were significantly downregulated by the YQHX treatment indicating that the mechanisms of YQHX might be involved in the inhibition of the TGF-*β* signaling pathway.

Apoptosis is a form of programmed cell death and plays an important role in maintaining the normal growth of organisms and eliminating aging and malignant cells in the body [24]. However, the process of apoptosis is precisely regulated in vivo, and whether insufficient or excessive apoptosis can both lead to the occurrence of related diseases such as cell fibrosis and necrosis [25, 26]. In particular, numerous research studies have suggested that apoptosis of renal cells may result in the development of renal fibrosis and cell death [27, 28]. In the process of the mitochondrial apoptotic pathway, abnormal expression of the antiapoptotic protein Bcl-2 can promote the release of cyto-C from cell mitochondria [29]. Thereafter, cyto-C combined with the apoptotic protein-activating factor 1 to form a complex, induces activation of the caspase family in a cascaded manner and eventually leads to cell apoptosis by a key apoptotic proteinase, caspase-3 [30]. Owing to the initiation and progress of apoptosis in the renal cells, the cell microenvironment was altered, causing the release of TGF-*β*, Ang-II, and other inflammatory mediators, which together triggered renal inflammation and fibrosis [31]. Thus, in the present study, we detected the apoptosis level of CKD rats and found that the overexpression of caspase-3 and decreased Bcl-2 level were reversed by the YQHX formula, which suggests that the beneficial effect of YQHX might be related to the regulation of apoptosis.

Autophagy has been reported as an important mechanism for maintaining cell homeostasis under conditions of cell stress, which is related closely to cell apoptosis and fibrosis [32, 33]. In recent years, mounting evidence has proved that autophagy is involved in the development of kidney disease, especially in renal fibrosis [34]. However, whether autophagy activation protects renal cells from injury remains controversial [9, 35]. It is reported that autophagy activation protects renal cells from injury induced by aristolochic acid, cisplatin, or cyclosporine A [36–38]. However, in the model of ischemia/reperfusion or tunicamycin-induced renal injury, increased autophagic activity contributes to TECs death [39, 40]. Therefore, autophagy activation plays a different role in different state of the kidney or the stress factors.

In our previous study [13], we detected the autophagy level in a high-grade proteinuria rat model developed by a cationic bovine serum albumin (C-BSA) injection, treated with or without rapamycin (an autophagy agonist). We found that the TECs of the model rats showed an increased expression level of microtubule-associated protein 1 light chain 3 (LC3)-II (a key maker of autophagosome), which were dramatically further upregulated in rapamycin-treated rats. Moreover, a lower p62 expression level and apoptosis were observed in the TECs of the rapamycin group, which indicated that autophagy response might be a protective mechanism in renal cells against injuries induced by urinary proteins.

**Figure 7:**
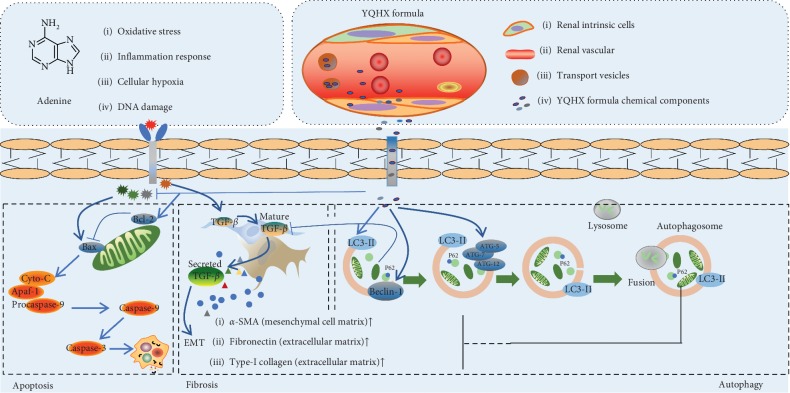
Schematic diagram of the effect of the YQHX formula on the renal tubular cell damage induced by adenine. Adenine feeding induced a series of injury factors, including oxidative stress, inflammation response, cellular hypoxia, and DNA damage, which further caused cell apoptosis by eventually activating caspase-3 and induced cell fibrosis by initiating the TGF-*β* signaling pathway. TGF-*β* signaling contributes to the phenotypic conversion program of the epithelial-mesenchymal transition (EMT) by gaining mesenchymal features (including *α*-smooth muscle actin, interstitial matrix components type I collagen, and fibronectin). The effective components of the YQHX formula ameliorated the cell apoptosis by upregulating the Bcl-2 expression and sequentially reducing the caspase-3 expression. Meanwhile, the YQHX formula activated the autophagy pathway by increasing the levels of Beclin-1, ATG-5, and LC3-II, thereby degrading the substrate p62, misfolded proteins, and injured cell organelles. Mature TGF-*β* induced by injury factors was reduced by the autophagy pathway.

In the present study, the adenine-induced kidney disease rat model, which was characterized by increased proteinuria and many diverse complications, was applied to examine the therapeutic effect of the YQHX formula. As the results showed, the expression pattern of LC3-II in the adenine rats was similar with the previous study, which was further increased by the YQHX treatment. While, the increased p62 expression in the adenine group was reduced by the YQHX treatment. Nevertheless, both the elevated expressions of LC3-II and p62 in the adenine group could be a result of degradation obstruction in downstream, such as lysosome injury or failed fusion of autophagosomes and lysosomes to form autolysosomes, which might have been repaired by the YQHX treatment. However, in another case, the autophagic induction from upstream evidenced by increased LC3-II might be a self-restoration effect of the adenine rats, trying to repair the injury during the last 6 weeks. But the limited autophagy activation was not enough to degrade the largely accumulated p62 induced by the adenine injury. While, in the YQHX group, p62 was degraded by the remarkably strengthened autophagic induction. Thus, the exact working point on autophagy induction or degradation needs a further research. To further investigate the cause of autophagic induction, we have evaluated it by detecting a key protein, Beclin-1, which contributes to autophagosome formation [41]. The expression pattern of Beclin-1 was exactly consistent with that of LC3-II. In addition, another autophagic induction-relative protein, ATG-5, was also tested and showed an uptrend in the YQHX group as compared with the adenine group. Therefore, we suggest that the YQHX formula might improve autophagy activation by inducing the expression of Beclin-1. The possible mechanisms of YQHX on kidney disease are summarized in Figure 7.

## 5. Conclusions

The YQHX formula improves renal function and has an antifibrosis effect in the kidney disease. Its renoprotective effect may be attributed to the activation of the autophagic pathway. Our findings suggest that an increase in autophagy flux induced by TCM might provide a promising novel approach for treating the kidney disease.

## Figures and Tables

**Table 1:** Different components of YQHX.

Chinese name	Latin name	Botanical plant name	Main ingredients e.g.	English name	Family	Part used	Dose (g)
Huang Qi	Radix Astragali seu Hedysari	*Astragalus membranaceus* (Fisch.) Bge. var mongholicus (Bge.) Hsiao	Astragaloside, astragalus polysaccharide	Milkvetch root	Leguminosae	Dried root	30
Chuan Niu Xi	Radix Cyathulae	*Cyathula officinalis* Kuan.	Cyasterone, sengosterone	Medicinal cyathula root	Amaranthaceae	Dried root	15
Zhi Shi	Fructus Aurantii Immaturus	*Citrus aurantium* L.	Flavonoid, volatile oils	Immature orange fruit	Rutaceae	Dried young fruit	15
Hu Zhang	Rhizoma Polygoni Cuspidati	*Polygonum cuspidatum* Sieb. et Zucc.	Resveratrol, emodin	Giant knotweed rhizome	Polygonaceae	Dried rhizome and root	15
San Leng	Rhizoma Sparganii	*Spaganium stoloniferum* Buch.-Ham.	Phenethyl alcohol, Quinol	Common buried rubber	Sparganiaceae	Dried tuber	12
E Zhu	Rhizoma Curcumae	*Curcuma phaeocaulis* Val.	Curzerenone, borneol	Zedoray rhizome	Zingiberaceae	Dried rhizome	12
Tu Bie Chong	Eupolyphaga Seu Steleophaga	*Eupolyphaga sinensis* Walker	Amino acids, fatty acids	Ground beetle	Corydidae	Dried body	12
Shui Zhi	Hirudo	*Hirudo nipponica* Whitman	Amino acids, hirudin	Leech	Hirudinidae	Dried body	3

**Table 2:** Semiquantitative criteria for renal and hepatic lesions.

Degree	Kidney lesions					Liver lesions
	*Glomerulosclerosis*	*Interstitial fibrosis*	*Arteriolosclerosis*	*Interstitial inflammation*	*Tubular dilatation*	*Portal inflammation*
0	Normal	≤5% renal cortex	No or little intimal incrassation	No or little inflammation	No dilatation	None
1	≤25% mesangial expansion/sclerosis	6∼25% renal cortex	Intimal incrassation leads to lumen stenosis ≤15%	10%∼25% renal cortex	≤25% renal cortex tubule	Mild, some/all portal areas
2	25%∼50% mesangial expansion/sclerosis	26%∼50% renal cortex	Increased intimal incrassation leads to lumen stenosis ≤25%	26%∼50% renal cortex	26∼50% renal cortex tubule	Moderate, some or all portal areas
3	≥50% mesangial expansion/sclerosis	>50% renal cortex	Severe intimal incrassation leads to lumen stenosis ≥50%	>50% renal cortex	≥50% renal cortex tubule	Moderate/marked, all portal area

**Table 3:** Effect of YQHX treatment on some histopathological parameters in adenine rats.

	Control	Adenine	YQHX
Kidney lesion			
*Glomerulosclerosis*	0.1 ± 0.02	1.5 ± 0.04^△^	1.3 ± 0.04^△###^
*Interstitial fibrosis*	0.0 ± 0.0	2.6 ± 0.6^△^	2.3 ± 0.6^△###^
*Arteriolosclerosis*	0.0 ± 0.0	2.4 ± 0.7^△^	2.1 ± 0.6^△##^
*Interstitial inflammation*	0.0 ± 0.0	2.8 ± 0.4^△^	2.0 ± 0.7^△###^
*Tubular dilatation*	0.0 ± 0.0	2.6 ± 0.5^△^	2.1 ± 0.6^△###^
Liver lesion			
*Portal inflammation*	0.1 ± 0.2	2.1 ± 0.5^△^	1.2 ± 0.4^△###^

Values in the table are means ± SD; ^△^, *p* < 0.001 vs. the control group;^##^, *p* < 0.01;^###^, *p* < 0.001 vs. the adenine group.

## Data Availability

The data used to support the findings of this study are available from the corresponding author upon request.

## Supplementary Materials

Supplementary Materials Supplementary Table 1: semiquantitative criteria for IHC analysis. Supplementary Figure 1: YQHX treatment reduced the expression of renal fibrosis-relative proteins. (a–c) The assessment results of immunohistochemistry staining showing increased protein levels of *α*-SMA, Col-I, and TGF-*β*1 in the adenine group, which were decreased by the YQHX treatment. The number of rats per group was 5, and 10 pictures were taken from each rat. ^*∗∗∗*^*p* < 0.001. Adenine group, adenine-induced kidney disease rat; YQHX, yiqihuoxue group.
