# Supplementary material for: Yiqihuoxue Formula Activates Autophagy and Offers Renoprotection in a Rat Model of Adenine-Induced Kidney Disease
Source: Evid Based Complement Alternat Med. 2019 Dec 24;2019:3423981. doi: 10.1155/2019/3423981 (PMC6948341; doi:10.1155/2019/3423981)
Supplement: Supplementary Materials — Supplementary Table 1: semiquantitative criteria for IHC analysis. Supplementary Figure 1: YQHX treatment reduced the expression of renal fibrosis-relative proteins. (a–c) The assessment results of immunohistochemistry staining showing increased protein levels of α-SMA, Col-I, and TGF-β1 in the adenine group, which were decreased by the YQHX treatment. The number of rats per group was 5, and 10 pictures were taken from each rat. ∗∗∗p < 0.001. Adenine group, adenine-induced kidney disease rat; YQHX, yiqihuoxue group. [file 3423981.f1.pdf]

Supplymentary Table 1. Semiquantitative criteria for IHC analysis

Degree	α-SMA	Col-1	TGF-β1
0	5% ≤ area of interstitial field	5% ≤ area of cortex region	5% ≤ area of cortex region
1	6% - 25% area of interstitial field	6% - 30% area of cortex region	6% - 30% area of cortex region
2	26% -50% area of interstitial field	31% - 60% area of cortex region	31% - 60% area of cortex region
3	≥ 51% area of interstitial field	≥ 61% area of cortex region	≥ 61% area of cortex region

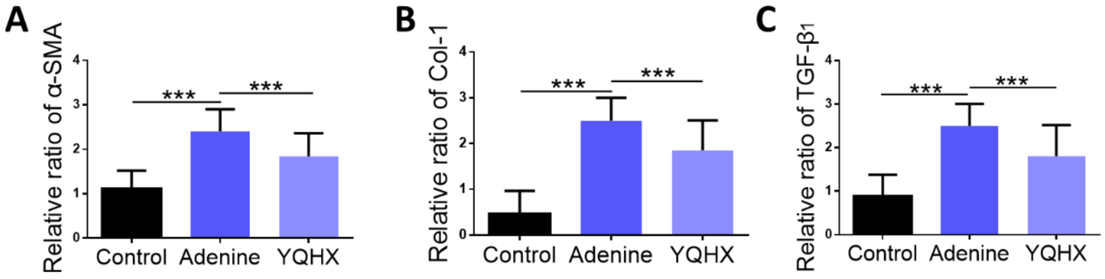

Supplymentary Figure. 1 YQHX treatment reduced the expression of renal fibrosis-relative proteins. (A–C) The assessment results of immunohistochemistry staining showing increased protein levels of α-SMA, Col-I, and TGF-β1 in the adenine group, which were decreased by the YQHX treatment. The number of rats per group was 5, and 10 pictures were taken from each rat. ***p < 0.001. Adenine group, adenine-induced kidney disease rat; YQHX, yiqihuoxue group.
